# Combination Treatment with Spacer Placement Surgery Followed by Particle Radiotherapy for Lymph Node Metastasis from Uterine Cancer

**DOI:** 10.1245/s10434-025-17039-9

**Published:** 2025-02-25

**Authors:** Shohei Komatsu, Satoshi Nagamata, Kazuki Terashima, Yusuke Demizu, Masaki Suga, Masahiro Kido, Hiroaki Yanagimoto, Hirochika Toyama, Sunao Tokumaru, Tomoaki Okimoto, Yoshito Terai, Takumi Fukumoto

**Affiliations:** 1 Department of Surgery, Division of Hepato-Biliary-Pancreatic Surgery, Kobe University Graduate School of Medicine, Kobe, Japan; 2 Department of Obstetrics and Gynecology, Kobe University Graduate School of Medicine, Kobe, Japan; 3 Department of Radiology, Hyogo Ion Beam Medical Center, Tatsuno, Japan; 4 Department of Radiation Oncology, Hyogo Ion Beam Medical Center Kobe Proton Center, Kobe, Japan; 5 Department of Radiation Physics, Hyogo Ion Beam Medical Center, Tatsuno, Japan

**Keywords:** Uterine cancer, Lymph node metastasis, Particle radiotherapy, Spacer placement surgery, Dose centralization

## Abstract

**Background:**

The effectiveness of local treatment in lymph node metastasis from uterine cancer has been proven; the standard treatment is surgical intervention. Although radiotherapy, including particle radiotherapy (PRT), is an alternative local treatment, its application is often contraindicated owing to its proximity to the gastrointestinal tract. Combination treatment with spacer placement surgery followed by PRT is a potential solution to this problem. This study aimed to evaluate the outcomes of this combination treatment of lymph node metastases from uterine cancer.

**Patients and Methods:**

Between December 2007 and March 2023, ten consecutive patients who underwent combination treatment comprising spacer placement surgery and subsequent PRT were assessed for treatment outcomes.

**Results:**

The median survival time was 53.5 months; the 3- and 5-year overall survival rates were 76.2% and 38.1%, respectively. The 3- and 5-year local control rates in all patients were both 88.9%. The median volume irradiated at 95% of the treatment planning dose (V95%) of the gross tumor volume, clinical target volume, and planning target volume were 100.0%, 99.8%, and 92.2%, respectively. The median dose intensity covering 95% of the target volume (D95%) of the gross tumor volume/planned dose, clinical target volume/planned dose, and planning target volume/planned dose were 98.9%, 99.0%, and 87.2%, respectively.

**Conclusions:**

Spacer placement surgery contributed to the optimized PRT dose distribution and might have contributed to favorable local control and survival rates. This innovative combination treatment might have a significant effect on the treatment of lymph node metastases from uterine cancers.

Historically, radiotherapy has been one of the most important treatment options for malignant tumors. Recently, the development of particle radiotherapy (PRT), such as proton and carbon ion therapy, has had a major influence on the field of radiotherapy and come to play an important role in the treatment of various malignant tumors.^1–3^ The use of PRT is increasing and has reached an important position in management guidelines for some tumors. The greatest advantage of PRT is its superior dose distribution, known as the Bragg peak.^4,5^ However, radiotherapy has a drawback related to its inability to radically irradiate tumors that are broadly adjacent to the gastrointestinal tract, owing to the low dose tolerance of the gastrointestinal tract. Even with the sophisticated dose distribution of PRT, curative doses cannot be delivered to entire tumors broadly adjacent to the gastrointestinal tract.^6^ To enable radical irradiation with PRT, a two-stage treatment referred to as space-making particle therapy (SMPT), consisting of the combination of spacer placement surgery followed by PRT, has been employed for such tumors.^6–8^ The clinical results of SMPT have been reported recently for several malignant tumors, and its usefulness is becoming well established.^9–11^

Uterine and endometrial cancers are two of the most common cancer types in women worldwide.^12,13^ Lymph node metastases represent the most frequent forms of recurrence, for which surgery is the first treatment option. In particular, regarding lymph node metastases in the pelvic and paraaortic regions, the guidelines recommend surgery, chemotherapy, or radiotherapy, which are currently selected on a case-by-case basis according to the treatment course leading up to the recurrence, site of recurrence, and patient and tumor factors. The effectiveness of radiotherapy as a locoregional treatment for lymph node metastases remains under investigation. A recent study has suggested a survival benefit in patients with metastatic disease who received pelvic radiotherapy; however, the heterogeneity of the patient population in that study did not allow for any definite conclusions.^14^ Although there have already been reports regarding the effectiveness of PRT in locally advanced uterine cancer,^15^ the number of reports is very small, and its position in the guidelines remains controversial. In addition, no reports have discussed the outcomes of SMPT for lymph node metastases from uterine cancers. This study aimed to evaluate the outcomes of SMPT for lymph node metastasis from uterine cancer.

## Patients and Methods

### Treatment Concept and Methods

**Fig. 1:**
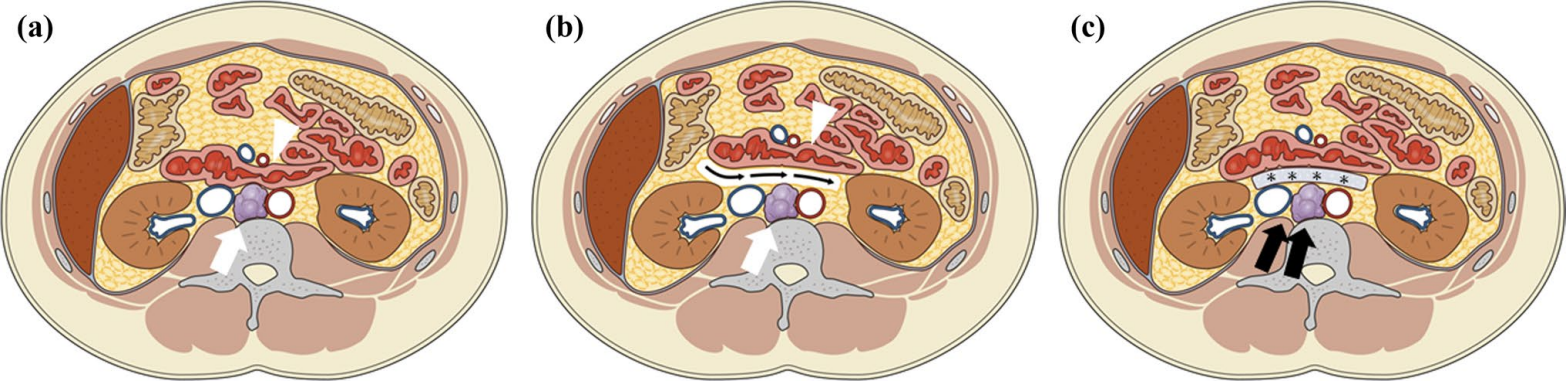
The concept of space-making particle therapy for lymph node metastasis from uterine cancer; **a** tumor (white arrow) is attached to the gastrointestinal tract (white arrowhead); **b** spacer placement surgery creates a space (black arrow) between tumor and the gastrointestinal tract; **c** owing to the space obtained by the spacer (asterisks), a radical dose of particle radiotherapy (black arrow) can be delivered from the dorsal side

The SMPT concept is shown in Fig. 1. Lymph node metastases from uterine cancers are generally located in the pelvic and paraaortic regions. Inevitably, lymph node metastases were adjacent to the nearby gastrointestinal tract (Fig. 1a), resulting in a lack of indications for curative radiotherapy. SMPT consisted of spacer placement surgery in the first stage (Fig. 1b) and subsequent PRT in the second stage (Fig. 1c). Owing to the sophisticated dose centralization of PRT, a 10-mm space between the tumor and the gastrointestinal tract is sufficient for radical irradiation. When tumors deeply invade the peritoneal side or adjacent structures and surgical resection margins are deemed to be impossible to secure, SMPT is usually selected. In such cases, spacer placement surgery is a preparatory step for PRT. Therefore, the tumor is never removed; rather, only the space between the tumor and the gastrointestinal tract is created. When tumors were determined to be resectable, such an attempt was made. When a positive surgical margin was suspected, spacer placement surgery was performed in preparation for postoperative PRT.

### Patients and Study Criteria

Between December 2007 and March 2023, ten consecutive patients underwent a combination treatment consisting of spacer placement surgery and subsequent PRT for lymph node metastases from uterine cancer. Spacer placement surgery was performed at the Kobe University Hospital, International Clinical Cancer Research Center, and Kobe Kaisei Hospital. Subsequent PRT was performed at the Hyogo Ion Beam Medical Center or Hyogo Ion Beam Medical Center Kobe Proton Center. The eligibility criteria for patient enrollment were as follows: (1) histologically or clinically diagnosed lymph node metastases from uterine cancer, (2) absence of metastases at sites other than the target tumors, (3) lymph node metastases that were broadly adjacent to the gastrointestinal tract and were deemed unsuitable for curative PRT, (4) World Health Organization performance status of 0–2, (5) adequate organ function, and (6) no active concomitant malignancy.

Written informed consent was obtained following a thorough explanation about the study, which was conducted in accordance with the ethical standards of the Declaration of Helsinki and approved by the ethics committees of Kobe University (approval no. B240041), International Clinical Cancer Research Center, Kobe Kaisei Hospital, Hyogo Ion Beam Medical Center, and Hyogo Ion Beam Medical Center Kobe Proton Center.

### Spacer Placement Surgery

The abdomen was opened through a median lower abdominal incision. After confirming the absence of peritoneal dissemination or metastatic lesions other than the target lesion, spacer placement surgery was performed. The procedure was performed to secure at least a 10-mm margin in all directions between tumor and the gastrointestinal tract. The absorbable polyglycolic acid (PGA) spacer, which was developed specifically as a spacer material, has been used since its approval for clinical use in 2019.^16,17^ The PGA spacer was 10 cm wide and 20 cm long, and it was available in three thicknesses: 5, 10, and 15 mm. The surgeons could trim the PGA spacer to an appropriate shape using a scalpel and scissors, depending on the intraoperative findings. The PGA spacer was superimposed between the tumor and the adjacent gastrointestinal tract and fixed tightly to the surrounding tissue with absorbable strings to avoid deviation. Prior to this, expanded polytetrafluoroethylene sheets or the greater omentum were used as spacer materials.^6–8,18^ Antibiotics were administered for at least 3 days to prevent postoperative infection.

### Particle Radiotherapy Treatment Protocol

The treatment plan was set by a computed tomography (CT)-based three-dimensional treatment planning system (Xio-M; Mitsubishi Electric Corporation, Tokyo, Japan). Each patient was immobilized with a custom-made thermoplastic cast in the supine position, and 2-mm-thick CT images were taken during the exhalation phase using a respiratory gating system. The nodal metastatic lesion was defined as the gross tumor volume (GTV). The clinical target volume (CTV) was set as the GTV plus a 5-mm margin in all directions. The planning target volume (PTV) was defined as the CTV plus a 5-mm setup margin with an appropriate internal margin under the respiratory gating system as necessary. Passive scattering techniques have been used for proton and carbon ion therapies, whereas active scanning techniques have only been available for proton therapy since 2020. Dose-volume histograms were calculated for all patients to assess the dose intensity and risk of radiation-induced adverse events. The treatment plan was developed to maximize the volume irradiated with 95% of the treatment planning dose (V95%) of the GTV, CTV, and PTV, while ensuring that the maximum scalp dose (Dmax) and irradiated volume of 0.5 cm^3^ of the gastrointestinal tract remained below the facility-specific dose limits (which varied depending on the number of fractions).

The treatment policy for selecting proton or carbon ion beams was based on tumor characteristics and dose distribution. Both facilities used a gantry for proton therapy, enabling irradiation from any angle. In contrast, carbon ion therapy could only be used to irradiate from fixed ports at 0°, 45°, and 90°. Although carbon ions could provide better dose concentration, the limited angles available might lead to a more favorable dose distribution with protons depending on the relative positioning of tumor and the organs at risk. Therefore, treatment plans for both proton and carbon ion therapies were created for managing all cases, and a selection was made on the basis of a comparison between their dose-volume histograms. The relative biological effectiveness (RBE) of protons was calculated as 1.1, while the RBE of carbons was determined on the basis of the RBE for skin reactions, which was assessed to be 3.0 at the distal part of the spread-out Bragg peak.

### Methods for Analyzing Post-space-making Particle Radiotherapy Dose-Distribution

Dosimetric evaluations were assessed by dose-volume histograms considering V95% and dose intensity covering 95% of the target volume (D95%) of the GTV, CTV, and PTV. The Dmax and irradiated volume of 2 cm^3^ (D2cc) values of the small intestine and colon were calculated to assess the irradiation doses to the gastrointestinal tract. These values are often used as parameters to evaluate the PRT dose centralization.

### Follow-Up and Statistical Analyses

The patients were followed up through physical examinations, laboratory data, CT, and magnetic resonance imaging every 3–4 months. Local recurrence after PRT was defined as the enlargement of tumors within the irradiation field on the basis of previously reported data.^19^ Complications of spacer placement surgery were assessed according to the Clavien–Dindo classification system.^20,21^ Moreover, adverse events associated with PRT were determined using the National Institute Common Terminology Criteria for Adverse Events version 5.0. Overall survival and local control rates were assessed using the Kaplan–Meier method and evaluated using log-rank tests. Statistical analyses were conducted using the JMP 17 statistical package (SAS Institute, Cary, NC, USA); statistical significance was set at *P* < 0.05.

## Results

### Patient Characteristics

The characteristics and treatment courses in all the patients treated with SMPT are presented in Table 1. The median patient age was 50 years (range 31–74 years). All patients were diagnosed with lymph node metastasis from uterine cancer. Of the ten patients, nine were diagnosed with cervical cancer recurrence and one was diagnosed with endometrial cancer. Regarding the recurrence site, there were nine recurrences in the pelvic region (internal and external iliac lymph nodes, obturator lymph nodes, and parametrial lymph nodes) and one in the paraaortic region. Regarding treatment history, nine of the ten patients had a history of hysterectomy for uterine cancer. Two patients underwent hysterectomy alone; five underwent hysterectomy plus systemic chemotherapy; two received hysterectomy, systemic chemotherapy, and radiotherapy; and one received radiotherapy and systemic chemotherapy. Regarding the spacer placement surgery, the median operative time was 165 min (135–325 min); the median blood loss was 30 mL (10–330 mL). Regarding the operative procedure, expanded polytetrafluoroethylene sheets placement and PGA spacer placement were performed in six and two patients, respectively. The remaining two patients underwent intestinal resection with omental implantation.

**Table 1:** Patient characteristics

Case	Age, years	Cancer type	LN location	Treatment course	Type of spacer	PRT protocol (Gy (RBE)/Fr)	Adverse events (surgery/PRT)
1	31	Cervical	Rt, pelvic	ope, chemo	ePTFE	Proton 50/25	Subileus (G2)/dermatitis (G1)
2	36	Cervical	Rt, pelvic	ope, chemo	ePTFE	Carbon 55.2/12	None/dermatitis (G1)
3	45	Cervical	Rt, pelvic	ope, chemo	ePTFE	Proton 64/16	UTI (G2)/dermatitis (G2)
4	47	Cervical	Lt, pelvic	ope, chemo,RT	ePTFE	Carbon 64/8	None/none
5	51	Cervical	Rt, pelvic	ope	PGA	Proton 72.6/22	Serum liver enzyme elevation (G1)/dermatitis (G1)
6	53	Cervical	Rt, pelvic	chemo, ope, chemo	Omentum*	Proton 64/8	None/bone fracture (G2)
7	57	Cervical	Rt, pelvic	ope, chemo,ope	Omentum*	Carbon 64/8	None/dermatitis (G1)
8	48	Cervical	Para-Aorta	ope, chemo, RT	PGA	Carbon 55.2/12	None/dermatitis (G1)
9	74	Endometrial	Rt, pelvic	ope	ePTFE	Proton 72.6/22	None/dermatitis (G2), neuropathy (G2), rectal bleeding (G3)
10	59	Cervical	Lt, pelvic	RT, chemo	ePTFE	Proton 66/33	Serum liver enzyme elevation (G2), UTI (G2)/dermatitis (G1)

*LN* lymph node, *PRT* particle radiotherapy, *Gy* gray, *RBE* relative biological effectiveness, *Fr* fraction, *Rt* right, *Lt* left, *ope* operation, *chemo* chemotherapy, *RT* radiotherapy, *ePTFE* expanded polytetrafluoroethylene sheet, *PGA* polyglycolic acid spacer, *G* grade, *UTI* urinary tract infection

*Intestinal resection with omental implantation

**Fig. 2:**
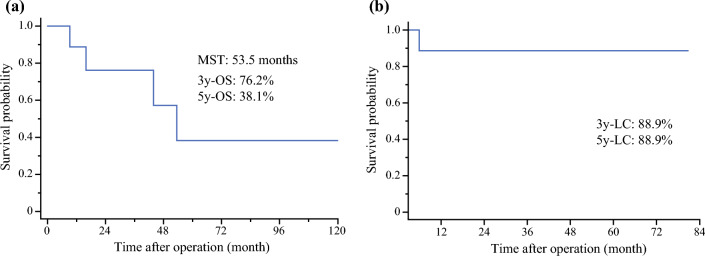
Overall survival (**a**) and local control rates (**b**) of all ten patients who underwent space-making particle therapy for lymph node metastasis from uterine cancer

Proton therapy was administered to six patients, while carbon ion therapy was delivered to four patients (Table 1). Among the ten patients, five died before the last follow-up, and the median follow-up period was 39.8 months. One patient did not undergo post-treatment imaging; thus, recurrence evaluation was not possible. Only one of the remaining nine patients experienced local recurrence. The median survival time of all patients was 53.5 months, and the 3- and 5-year overall survival rates were 76.2% and 38.1%, respectively (Fig. 2a). The 3- and 5-year local control rates in all patients were 88.9% (Fig. 2b).

**Fig. 3:**
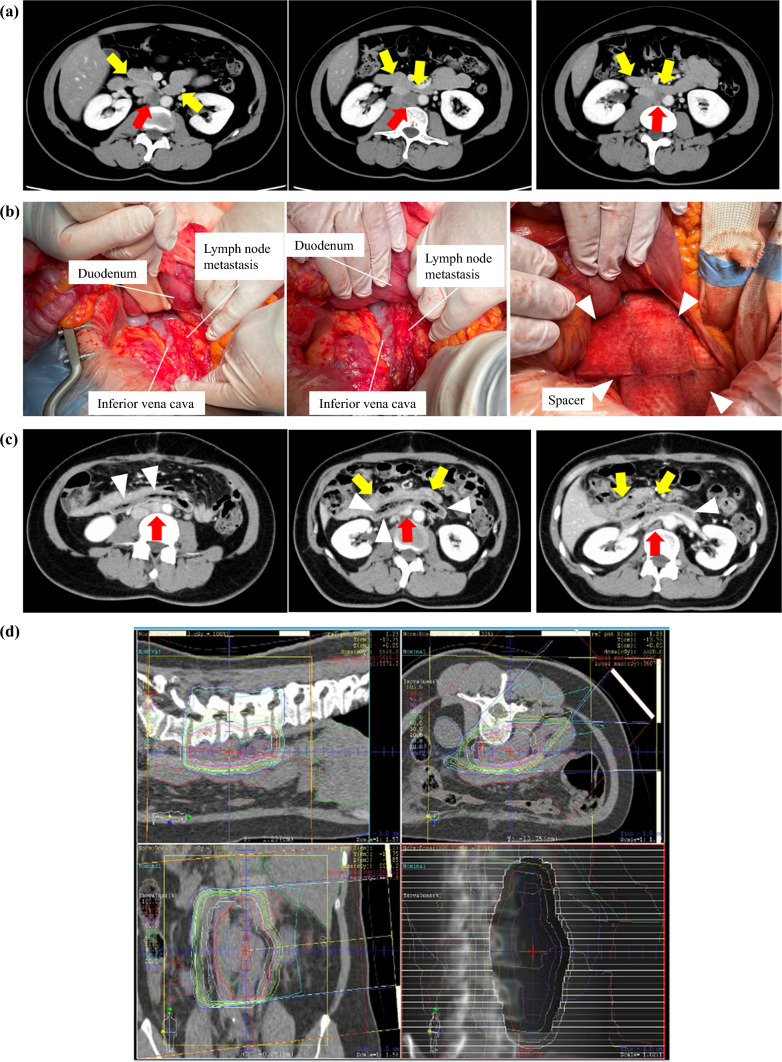
Representative case undergoing space-making particle therapy for lymph node metastasis from uterine cancer in a paraaortic lesion; **a** abdominal computed tomography before spacer placement surgery showing that the lymph node metastasis (red arrow) between the abdominal aorta and inferior vena cava abuts the duodenum (yellow arrow); **b** intraoperative findings; after Kocher’s maneuver, the lymph node metastasis located on the left side of the inferior vena cava was dissected from the duodenum; a polyglycolic acid spacer (white arrowhead) was placed between the tumor and the duodenum; **c** postoperative abdominal computed tomography revealing that the polyglycolic acid spacer (white arrowhead) kept sufficient space between the lymph node metastasis (red arrow) and the duodenum (yellow arrow); **d** carbon ion therapy with 55.2 gray (relative biological effectiveness) in 12 fractions was delivered; the V95% (volume irradiated with 95% of the treatment planning dose) of the gross tumor volume and clinical target volume were 100.0% and 99.4%, respectively; the D95% (dose intensity covering 95% of the target volume) of the gross tumor volume/planned dose and clinical target volume/planned dose were 98.9% and 98.8%, respectively; D2cc of the duodenum was 2.6 gray (relative biological effectiveness)

Figure 3 shows a representative case of lymph node metastasis in a patient with uterine cancer receiving SMPT. Preoperative abdominal CT (Fig. 3a) revealed lymph node metastasis in the paraaortic region (red arrow), which was widely attached to the duodenum and small intestine (yellow arrow). After performing Kocher’s maneuver, the duodenum was detached from the inferior vena cava, and the PGA spacer (white arrowhead) was placed and tightly sutured to the surrounding tissue between the lymph node metastasis and the duodenum (Fig. 3b). Postoperative CT revealed that the PGA spacer maintained sufficient space between the lymph node metastasis (red arrow) and the duodenum (yellow arrow) (Fig. 3c). Owing to the space created by the PGA spacer, a sufficient curative dose could be delivered to the tumor, and sophisticated irradiation with GTV V95% and CTV V95% of 100% and 99.4%, respectively, was delivered. The D2cc of the duodenum was 2.6 gray (Gy) (RBE) (Fig. 3d).

### Complications

Complications related to surgical spacer placement were observed in four patients (40%). No severe complications (grade 3 or higher) were observed. Complications were classified as follows: grade 2 urinary tract infection in two patients, grade 2 subileus in one patient, and elevation in liver enzyme levels in two patients (grade 1 and 2, one each). Regarding the PRT complications, acute complications were seen in seven patients (70%) and all were grade 1 or 2 dermatitis (grade 1 in five patients and grade 2 in two patients). Late complications were observed in five patients: dermatitis (grade 1 in three patients and grade 2 in one patient), grade 2 bone fracture in one patient, grade 2 peripheral neuropathy in one patient, and grade 3 rectal bleeding in one patient.

### Dosimetric Evaluation of Particle Radiotherapy

Dose analyses based on the dose-volume histogram are summarized in Table 2. The median V95% values of the GTV, CTV, and PTV were 100.0%, 99.8%, and 92.2%, respectively. The D95% values of the GTV/planned, CTV/planned, and PTV/planned doses were 98.9%, 99.0%, and 87.2%, respectively. V95% and D95% are commonly used indices for evaluating dose concentration in the planning of radiotherapy, and the fact that all values of V95% of the GTV and CTV and D95% of the GTV/planned dose and CTV/planned dose exceeded 95% indicated a favorable dose distribution in the present series. Of the ten cases, the dose distributions were poor in two cases (case 1 and case 10, Table 1). Local recurrence developed in case 10, and radiological evaluation after PRT could not be performed for case 1. The V95% of the GTV for these two cases were 68.6% and 76.3%, respectively. In the remaining eight patients without local recurrence, the median V95% of the GTV, CTV, and PTV were 98.8% (range 91.6–100%), 98.4% (range 87.9–100%), and 92.9% (range 80.7–99.4%), respectively.

**Table 2:** Dosimetric evaluation by dose volume histogram parameters

Parameter	Value
V95% of GTV (%)	100.0 (68.6–100.0)
V95% of CTV (%)	99.8 (60.1–100.0)
V95% of PTV (%)	95.1 (41.7-99.9)
D95% (Gy [RBE]) of GTV/planned dose (%)	98.9 (73.5–99.8)
D95% (Gy [RBE]) of CTV/planned dose (%)	99.0 (55.0–99.7)
D95% (Gy [RBE]) of PTV/planned dose (%)	87.2 (22.3–98.7)
Dmax of small intestine (Gy [RBE])	12.0 (1.1–46.5)
D2cc of small intestine (Gy [RBE])	4.0 (0.2–21.3)
Dmax of colon (Gy [RBE])	18.9 (2.3–74.2)
D2cc of colon (Gy [RBE])	7.1 (0.1–73.2)

Data are presented as median (interquartile range)

*GTV* gross tumor volume, *CTV* clinical target volume, *PTV* planning target volume, *V95%* volume irradiated with 95% of the treatment planning dose, *D95%* dose intensity covering 95% of the target volume, *RBE* relative biological effectiveness, *Dmax* maximum scalp dose, *D2cc* irradiated volume of 2 cm^3^

The median Dmax and D2cc of the small intestine were 12.0 Gy (RBE) (range 1.1–46.5) and 4.0 Gy (RBE) (range 0.2–21.3), respectively. The median Dmax and D2cc of colon were 18.9 Gy (RBE) (range 2.3–74.2) and 7.1 Gy (RBE) (range 0.1–73.2), respectively.

### Specific Case Series

#### Case of Local Recurrence (Case 10)

The V95% of the GTV, CTV, and PTV were quite low in only one local recurrence case at 76.3%, 60.9%, and 41.7%, respectively (case 10 in Table 1). In this case, a sufficient spacer was not inserted up to the lower rectum, resulting in the GTV being in close proximity to the rectum. The CTV, which included a 5-mm margin around the GTV, came into contact with the rectum, leading to inadequate coverage.

#### Cases of Re-irradiation (Case 4, Case 8, and Case 10)

In the present study, three cases underwent re-irradiation of PRT after photon radiotherapy (cases 4, 8, and 10 in Table 1). In case 4 and case 10, photon radiotherapy with 50.4 Gy in 28 fractions (case 4) and combination of photon radiotherapy with 50.4 Gy in 28 fractions and brachytherapy with 18 Gy in 3 fractions (case 10) were delivered to the pelvic area before SMPT. Radical irradiation with PRT was performed to the extent possible, taking into account the tolerable dose from the previous treatment plan.

In case 8, carbon-ion therapy with 55.2 Gy (RBE) in 12 fractions was administered to treat the paraaortic lymph node metastases, located cranially to the area previously irradiated with 50.4 Gy in 28 fractions during the prior radiation therapy. The small bowel, irradiated in the previous treatment, was spared during this PRT owing to the use of a surgical spacer. The cauda equina, which was exposed to radiation during the previous treatment, was completely shielded from the carbon ion beams by adjusting the beam direction.

In all cases, there was no effect of previous photon radiotherapy at the time of spacer placement surgery; the procedure could be performed safely. Regarding side effects, the postoperative course was also uneventful.

#### Case of Rectal Bleeding (Case 9)

Complication of grade 3 or higher was observed only in one case of rectal bleeding (case 9 in Table 1). The Dmax and D2cc of the rectum in the case of rectal bleeding were 74.2 and 73.2 Gy (RBE), respectively. The V95% of the GTV and D95% of the GTV/planned dose were 100% and 99.8%, respectively, with quite favorable dose distribution. In this case, the dose plan was set up in a manner that did not comply with the appropriate dose constraints for the rectum, resulting in the delivery of significantly higher doses to the rectum, which was the cause of radiation proctitis.

## Discussion

Surgery, chemotherapy, and radiotherapy have been reported to be effective in the treatment of uterine cancer recurrence, and all these treatments are currently available depending on the recurrence type. Surgery and radiotherapy are often the first-line treatments of localized recurrence owing to their curability. PRT is one of the most sophisticated forms of radiotherapy; its usefulness for uterine cancer has been reported in recent years.^15,22^ However, PRT is still in its infancy, and there are few reports proving its effectiveness. Okonogi et al.^23^ identified the dose–volume relationships in PRT regarding the occurrence of late morbidities in nearby organs at risk, such as the rectum and bladder. The difficulties in using PRT in the pelvic region, where the gastrointestinal tract is in close proximity, have already been discussed. The present study demonstrated the clinical effectiveness of SMPT for lymph node metastases from uterine cancer. Spacer placement surgery was feasible, and dosimetric evaluation revealed that a sufficient curative dose of PRT could be delivered to the tumor in most cases. The favorable dose centralization represented by the dosimetric parameters (Table 2) may contribute to a high local tumor control rate, resulting in a prolonged survival prognosis, indicating the validity of SMPT for lymph node metastases from uterine cancer. Although local recurrence was observed in one case of poor dose distribution, none of the cases of a favorable dose distribution with a V95% of the GTV of more than 90% showed local recurrence. To our knowledge, this is the first study to demonstrate the effectiveness of combination therapy with spacer placement surgery and subsequent PRT in patients with lymph node metastases from uterine cancer. Although the number of cases in the present study was small and future studies are warranted, the clinical significance of PRT for the treatment of recurrent uterine cancer will be profound once it is established.

Although positive surgical resection margins have been reported to have a negative impact on recurrence and prognosis,^24^ securing a sufficient surgical margin is often difficult with any type of surgical procedure, particularly for tumors that broadly or deeply invade the retroperitoneum and/or large vessels. PRT can provide sufficient doses to tumors that have invaded or are in contact with vessels and provide sufficient margins to the vertical and horizontal axes for deeply invaded tumors as well. Previous reports have demonstrated equal effectiveness regarding local control rates after PRT, even for some tumor types which deeply invaded or were in contact with blood vessels.^25^ Thus, PRT has the potential for overcoming the limitations of surgery, and its effective use may lead to significant clinical advantages. Spacer placement surgery has made a tremendous contribution to expanding the curative treatment range of PRT by eliminating its greatest weakness: the inability to radically irradiate cases in close proximity to the gastrointestinal tract. The feasibility and effectiveness of the newly developed PGA spacer made of absorbable materials has been shown, and this combination treatment may be an innovative treatment option.^26^

Although infrequent, some malignant tumors may benefit from local treatment of aortic lymph node metastases. The area surrounded by the aorta and inferior vena cava is often associated with multiple technical surgical constraints. Considering complete tumor resection in this area, securing sufficient surgical margins can sometimes be difficult owing to the complex anatomical structures. As shown in the present case (Fig. 3), dissection of the duodenum from the peritoneum, including the inferior vena cava and abdominal aorta, using Kocher’s maneuver is not technically challenging, and subsequent radical PRT may potentially achieve a therapeutic effect comparable to that of R0 surgery. With appropriate spacer placement, irradiating areas more extensively than those that can be surgically removed is possible, which may be an effective treatment option in suitable cases. However, two cases of local recurrence and rectal bleeding were included wherein the effect of spacer placement surgery seemed inadequate. It is apparent that effective spacer placement surgery, if possible, might contribute to local control and prolonged survival; the quality of appropriate spacer placement surgery is required.

The limitations of the present study include its retrospective design, the small number of patients, and patient selection bias. Although further analyses with a larger number of patients are required, this is the first report to assess the effectiveness of SMPT for lymph node metastases of uterine cancer. In conclusion, SMPT might be a valid treatment option for lymph node metastases from uterine cancer.
